# Announcement

**Published:** 1913-09

**Authors:** W. A. Guild

**Affiliations:** Suite 230, The Utica.; Des Moines, Iowa


### Announcement.

THE ANNUAL CONVENTION AND CLINIC OF THE AMERICAN ASSOCIATION OF ORIFICIAL SURGEONS, CHICAGO, SEPTEMBER 23D TO 26TH, INCLUSIVE.

All physicians of all schools are invited to attend both. Dr. E. H. Pratt, father of Orificial Surgery, will personally operate and lecture. The Clinic will be under the supervision of a faculty of teachers composed of prominent orificial surgeons. At the clinic surgical methods of cure for all chronic diseases will be demonstrated daily: Asthma, dyspepsia, paralysis, eczema, and rheumatism, as well as diseases that present simply an aggravated attack of local pathology will be treated. It will pay you, doctor, to attend. Surgeons and general practitioners throughout the country testify that orificial surgery applied in everyday practice has added thousands of dollars to their incomes and immensely to their reputations for curing chronics.

As you may know, orificial surgery applies particularly to the freeing of sympathetic nerve impingements at the orifices of the body. Those attending will be taught to recognize and correct orificial defects.

The clinic is free. The Association will look after all details in an endeavor to broaden the scope of this great therapeutic measure. The clinic will be held in the amphitheater of the Francis E. Willard Hospital, 710 South Lincoln Street, across from the Cook County Hospital.

In the convention papers will be read by prominent men upon the Philosophy of Orificial Surgery, its technique, its application in the treatment of refractory chronic ailments. The headquarters for the convention will be at the Hotel La Salle, La Salle at Madison Street. All its sessions, programs and committee meetings will be held therein.

For further particulars you are invited to address the Secretary,

W. A. Guild, M. S., M. D.,

Suite 230, The Utica. Des Moines, Iowa.

---

Vein-to-vein transfusion possesses over the artery--to-vein operation at least the advantage of sparing the donor a conspicuous scar and the loss of a large artery. With a tourniquet lightly applied to his arm the venous pressure may be made abundant, and the blood flow correspondingly rapid.—*American Journal of Surgery.*

